# Clonal competition in BcrAbl-driven leukemia: how transplantations can accelerate clonal conversion

**DOI:** 10.1186/s12943-017-0668-x

**Published:** 2017-07-14

**Authors:** Kerstin Cornils, Lars Thielecke, Doreen Winkelmann, Tim Aranyossy, Mathias Lesche, Andreas Dahl, Ingo Roeder, Boris Fehse, Ingmar Glauche

**Affiliations:** 1 Research Department Cell and Gene Therapy, Department of Stem Cell Transplantation, University Medical Center Hamburg-Eppendorf, Hamburg, Germany; 2 Institute for Medical Informatics and Biometry, Faculty of Medicine Carl Gustav Carus, Technische Universität Dresden, Dresden, Germany; 3 Deep Sequencing Group SFB 655, Biotechnology Center, Technische Universität Dresden, Dresden, Germany; 4 Present Adress: University Medical Center Hamburg-Eppendorf, Pediatric Hematology and Oncology & Research Institute Children’s Cancer Center Hamburg, Martinistr. 52, 20246 Hamburg, Germany

**Keywords:** BcrAbl, Genetic barcodes, Leukemia, Heterogeneity, Clonal dynamics, Mathematical modelling, Clonal competition

## Abstract

**Background:**

Clonal competition in cancer describes the process in which the progeny of a cell clone supersedes or succumbs to other competing clones due to differences in their functional characteristics, mostly based on subsequently acquired mutations. Even though the patterns of those mutations are well explored in many tumors, the dynamical process of clonal selection is underexposed.

**Methods:**

We studied the dynamics of clonal competition in a BcrAbl-induced leukemia using a γ-retroviral vector library encoding the oncogene in conjunction with genetic barcodes. To this end, we studied the growth dynamics of transduced cells on the clonal level both in vitro and in vivo in transplanted mice.

**Results:**

While we detected moderate changes in clonal abundancies in vitro, we observed monoclonal leukemias in 6/30 mice after transplantation, which intriguingly were caused by only two different BcrAbl clones. To analyze the success of these clones, we applied a mathematical model of hematopoietic tissue maintenance, which indicated that a differential engraftment capacity of these two dominant clones provides a possible explanation of our observations. These findings were further supported by additional transplantation experiments and increased BcrAbl transcript levels in both clones.

**Conclusion:**

Our findings show that clonal competition is not an absolute process based on mutations, but highly dependent on selection mechanisms in a given environmental context.

**Electronic supplementary material:**

The online version of this article (doi:10.1186/s12943-017-0668-x) contains supplementary material, which is available to authorized users.

## Background

Genome instability resulting in increasing intratumoral heterogeneity is one of the enabling characteristics of cancer [1]. In consequence, acquired intrinsic selective advantages may result in the dominance of the most competitive malignant clones under the given conditions. Furthermore, both intracellular and extracellular factors influence tumor growth and intratumoral clonal competition, including the (dysregulated) epigenome and the local microenvironment [2–4]. Occurrence of resistances is a typical example of such setting, in which an initially small, but preexisting and therapy-resistant tumor clone might only outgrow under a therapy that targets the non-resistant part of the tumor [5, 6].

Deep sequencing of individual cells from tumor samples to decipher their mutational load has fostered the understanding of clonal evolution during cancer progression. It allows to retrospectively identify the most competitive cell clones and to estimate the order of genomic alterations. Despite these successes, the precise timing of mutation occurrence is hardly reconstructable. Moreover, an accurate, retrospective estimation of the *clonal dynamics* of newly evolving, but successively outcompeted cell clones is difficult to obtain. However, analysis of inferior clones is necessary to understand tumor progression as a temporally extended, dynamic competition process of diverging cell clones.

Alternative approaches for studying clonal dynamics aim at prospectively marking individual cell clones (e.g., by using integrating viral vectors) and studying temporal development by tracking the viral integration sites (IS) or genetic barcodes delivered by the viral vector (see e.g. [7–12]). Such marking strategies are not able to reconstruct the emergence of newly mutated cell clones, because the marking event occurs before the novel mutation. However, they qualify to mark an already heterogeneous population, to investigate the temporal development of distinct clones, e.g. under different culture conditions, and thereby to study the inter-clonal competition process [13]. To investigate the clonal interplay during leukemic development, we and others explored the option to combine genetic barcodes with a leukemia-driving oncogene [14, 15].

Chronic myeloid leukemia (CML) is a hematologic disorder, characterized by increased myeloid cell counts in peripheral blood. CML is one of the few cancer entities, in which the driving mutation is clearly defined by a genomic translocation of chromosome 9 and 22, in which the 5′-end of Bcr is fused to the 3′-end of the proto-oncogene Abl1. The resulting fusion protein BcrAbl is a constitutively active protein tyrosine kinase, which leads to unimpeded cell proliferation [16]. The BcrAbl oncogene is the sole driving force to initially transform affected cells both in vitro and in vivo [17–19].

We made use of this oncogenic potential of BcrAbl to study clonal developments of leukemia in vitro and in vivo. In particular, we applied γ-retroviral vectors to both, deliver the BcrAbl oncogene and to unambiguously mark modified cells with a genetic barcode. We transduced IL-3-dependend Ba/F3 cells, which can be maintained in vitro but also transplanted into mouse recipients. We thus investigated whether the initially polyclonal composition of differently marked, BcrAbl-expressing cell clones was retained in vitro and during development of a murine CML-like disease in vivo. Finally, we molecularly characterized dominant clones from diseased mice.

Our study provides insights in the clonal dynamics resulting from intratumoral heterogeneity *beyond* mutational differences and into the different behaviors of leukemic cells both in vitro and in vivo.

## Methods

### Cloning of the BcrAbl barcode vector library and production of viral particles

The p210 BcrAbl wildtype construct [20] was cloned into the γ-retroviral vector MP91-IRES-eGFP [21, 22] together with the eGFP marker. The BC32-construct as described in Thielecke et al. [23] was introduced into the viral backbone by Gibson assembly [24]. The plasmid library was used for production of eco-pseudotyped retroviral particles as described [25].

### In vitro testing of vector construct

**Fig. 1:**
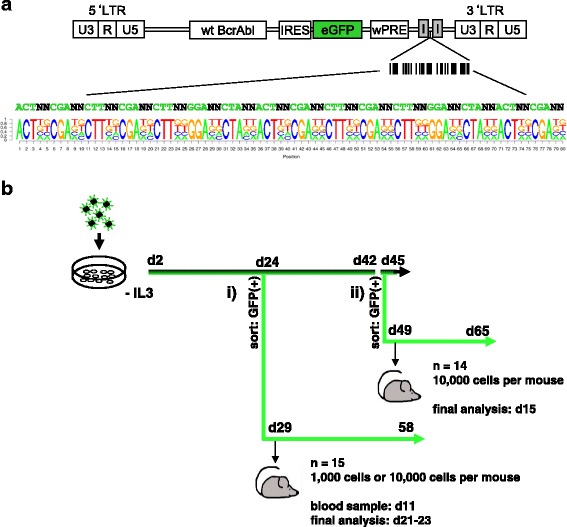
Vector construction and experimental set-up. **a** Wildtype BcrAbl (p210) in conjunction with eGFP was cloned into the γ-retroviral vector MP91 [21, 22]. The viral backbone was additionally equipped with a truncated sequence of Illumina Adaptors and our BC32 construct [23]. 10^10^ plasmids of the obtained plasmid library were used for next-generation sequencing via Miseq (Illumina) and >80,000 different plasmids were obtained. **b** BcrAbl-barcode containing viral particles were used to transduce the IL-3-dependent murine hematopoietic cell line Ba/F3. After IL-3 withdrawal, the cells were kept in culture and every 2–4 days an aliquot of cells was analysed by FC and used for DNA extraction. Cells were sorted for eGFP on day 24 (i) and on day 45 (ii) (after thawing). eGFP-positive cells were taken and transplanted into non-conditioned female Balb/C mice on day 29 (i) (1000 or 10,000 cells per mouse) and on day 49 (ii) (10,000 cells per mouse)

BcrAbl-BC vectors were used for transduction of Ba/F3 cells, which are strongly IL-3 dependent murine hematopoietic cells and initially described as B-cell precursors [26] to induce factor independence [27]. Analyses were performed in triplicates. Three days after transduction, samples were analyzed for eGFP expression and split, one half was still kept in IL-3-containing medium, the other was washed and seeded in medium without IL-3. Cells were passaged every 2 to 3 days, eGFP expression was measured via flow cytometry (FC) (LSR Fortessa, BD Bioscience, San Diego, CA, USA). Cell samples of every passage were taken for DNA extraction over a period of 45 days (including one freeze-thawing process). On day 25 and on day 45, a portion of the mixed population was sorted into eGFP-positive and eGFP-negative cells (Aria IIIu, BD Bioscience) (Fig. 1).

### In vivo experiments

On day 29, 1000 or 10,000 cells from the eGFP-positive culture were transplanted into immune-competent, non-conditioned Balb/C mice (*n* = 15 each). Blood samples were taken on day 11 after transplantation. On days 21–23, when some mice showed symptoms of leukemia, all mice were sacrificed and hematopoietic organs [peripheral blood (PB), bone marrow (BM) and spleen (Spl)] were isolated. After erylysis, cells were used for FC analysis and DNA extraction.

Another transplantation experiment was performed with cells cultivated in vitro for 49 days after transduction. Fourteen recipient mice were transplanted with 10,000 eGFP-positive cells each. On day 15, all mice showed signs of leukemia and were sacrificed for analysis as described before. Additionally, cells from spleens were used for expansion in vitro.

### Next-generation sequencing and bioinformatical analyses

10^10^ plasmids of the plasmid library or 200ng of genomic DNA (representing approx. 30,000 genomic equivalents) were subjected to PCR. Shortly, barcodes were amplified and Illumina-TruSeq Adaptor sequences were directly added (including Illumina based sample tags) in a single PCR step (Multiplex Plus Kit, Qiagen, Germany). Pooled libraries were quality-checked by capillar electrophoresis (Fragment Analyzer, Advanced Analytical, Ankeny, IA, USA) and sequenced with 83bp single reads on a Miseq (Illumina, San Diego, CA, USA). NGS-derived FASTQ-files were quality-filtered according to the associated Phred-score (accepted Phred-score>30). Barcode sequences were subsequently extracted and processed applying a newly developed error-correction method [23].

### Integration site analysis

Ligation-mediated (LM)-PCR was performed as described earlier [28]. Obtained sequences were analyzed using the MAVRIC online Tool [29].

### Transcript levels and RNA sequencing

In vitro cultured spleen cells from leukemic mice and as controls, sorted BcrAbl-transduced Ba/F3-bulk cells and untransduced Ba/F3 cells were subjected to RNA extraction (RNA Mini Kit, Qiagen) and subsequent cDNA synthesis (First strand cDNA Synthesis Kit, Thermo Fisher Scientific, Waltham, MA, USA). To determine transcript levels of BcrAbl in the respective clones, TaqMan-based real-time duplex PCR was performed with *Gapdh* as reference gene (Additional file 1: Table S3). For quantification, 10^1^–10^6^ copies of a clinically approved plasmid standard for BcrAbl [30] were measured in relation to a *Gapdh* plasmid standard (10^2^–10^8^ copies, Dharmacon Inc., Lafayette, CO, USA).

RNA samples were subjected to the standard workflow for strand-specific RNA-Seq library preparation (Ultra Directional RNA Library Prep, NEB) and were sequenced on an Illumina HiSeq 2500. Basic quality control of the reads was performed using FastQC (http://www.bioinformatics.babraham.ac.uk/). In addition, library diversity was assessed by redundancy investigation. Alignment of the reads to the mouse reference (mm10) was done with GSNAP (v2014–12-17) [31]. Ensembl gene annotation version 75 was used to detect splice sites. Uniquely aligned reads were counted with featureCounts (v1.4.6) [32] and the same Ensembl annotation. Normalization of the raw read-counts based on the library size and testing for differential expression between the three conditions (A, B and BcrAbl_bulk) was performed with the DESeq2 R-package (v 1.8.1) [33]. Genes with an adjusted *p*-value of less than 0.1 were considered as differentially expressed. KEGG gene set enrichment analysis was done with the clusterProfiler R-package [34].

### Simulations

We utilized an established single-cell based model framework for murine and human hematopoiesis [35–39]. For cells of all relevant clones (A, B, BcrAbl_bulk) we assumed a distribution of their engraftment capability (encoded by their initial attachment affinity), reflecting the fact that clone A and B have an increased probability for engraftment. From these distributions we sampled the corresponding number of cells that were observed in the analysis of the sequenced graft and ﻿used those to﻿ initialize the single-cell based model. In contrast to the host cells (which were initialized with the standard parameter set [38]), transplanted leukemic cells had an overall competitive advantage (i.e. increased proliferation rate encoded by altered transition functions, Additional file 1: Table S2). Every in silico transplantation was carried out mimicking an unconditioned host. Engraftment response was calculated as average over 250 transplantations. The second set of transplantations followed the same procedure only with adjusted cell numbers for the leukemic clones.

## Results

### BcrAbl-barcode vector transformed Ba/F3 cells to IL-3 independency in vitro

We generated a γ-retroviral plasmid vector library with BcrAbl and eGFP in conjunction with our BC32 barcode [23] that contained >80,000 different barcodes (Fig. 1a). Transduction of 100,000 Ba/F3 cells with viral particles derived from this library resulted in 2.5% eGFP-positive cells with a high probability for unique integrations. This initial transduction level declined slightly over time in the presence of IL-3, whereas cytokine deprivation led to a rapid increase to 60% eGFP-positive cells, followed by a slow decrease to 50% after 45 days (Additional file 1: Figure S1A). Prior to transplantation, we sorted the eGFP-positive cells from the BcrAbl_bulk culture on day 25 and on day 45.

### Clonal marking reveals polyclonal dynamics in vitro

NGS of freshly transduced Ba/F3 cells revealed 158 different barcodes, of which 148 barcodes (93.7%) were previously detected within the >80,000 barcodes from the plasmid library (Additional file 1: Figure S2). The initial number declined to 114 barcodes during in vitro culture (Additional file 1: Figure S1B). We also observed variability in clone sizes between consecutive time points. As the overall change in clonal abundances appeared modest, we suspect that various measurement errors further affected quantification. Fluctuations may have resulted from sampling effects during cell culture as well. We observed more pronounced changes in the clonal dynamics after sorting eGFP-positive cells. In fact, we detected a drop in the overall number of clones (probably due to the loss of eGFP-negative, but vector-positive clones), and an increase in the relative contribution of single clones (Additional file 1: Figure S3).

### In vivo tumorigenicity drives monoclonality in developing leukemias

**Fig. 2:**
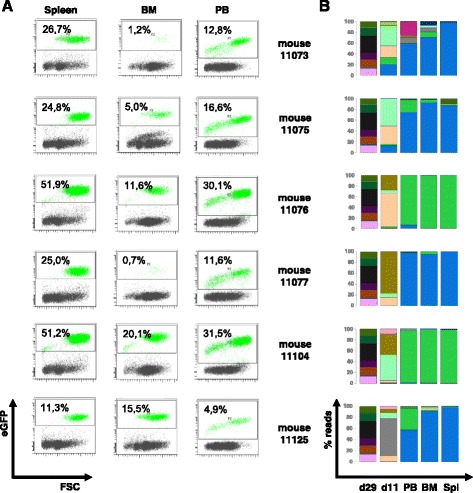
Final Analysis of the 1st mouse experiment. **a** eGFP expression in different hematopoietic organs (peripheral blood (PB), bone marrow (BM) and spleen (Spl)) at final analysis on day 21 or 23 post transplantation in the six mice which developed leukemia. **b** Distribution of BcrAbl-containing clones in the graft (d29), in the intermediate blood sample of day 11 post transplantation (d11) and in the hematopoietic organs. Genomic DNA was extracted from samples and used for PCR-amplification of the barcode-containing sequences. Sequencing was performed on a Miseq-Instrument and obtained sequences were analyzed by a customized R-script

We transplanted either 1000 (*n* = 15 each) or 10,000 eGFP-positive cells (*n* = 15 each) to address a potential impact of cell numbers. When six mice from the second group showed signs of leukemia (on days 21–23 after transplantation), all mice were sacrificed in accordance with the experimental setup. Diseased mice had enlarged spleens (Additional file 1: Table S1 and Figure S4), and eGFP-positive cells were detected in PB, BM and spleen (Fig. 2a). In all other mice, no signs of leukemia were observed, and no eGFP-positive cells were detected. We isolated DNA from PB, BM and spleen of leukemic mice for barcode amplification and NGS (Fig. 2b). We identified only one dominant clone per animal responsible for the leukemic outgrowth and surprisingly, only two different clones in total. Leukemia in two mice resulted from one clone (termed “A”, marked in green); the other four mice were dominated by a second clone (“B”, marked in blue). In the original graft (day 29), these two clones were minor, only 0.47% of all reads contained barcode of clone A, whereas 0.65% contained barcode of clone B.

**Fig. 3:**
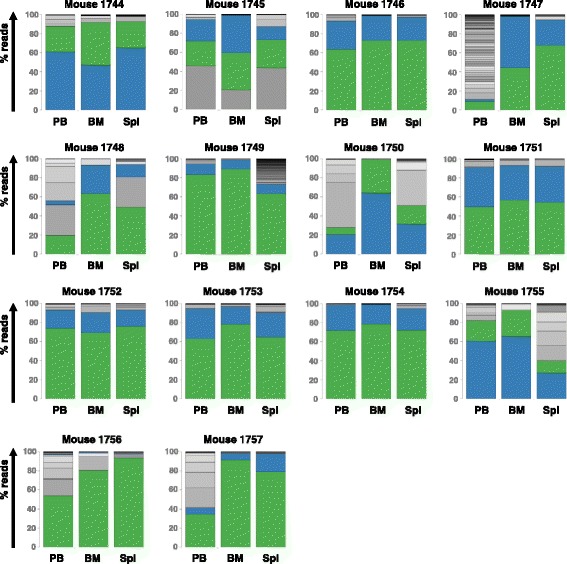
Final Analysis of the 2nd mouse experiment. Distribution of BcrAbl-containing clones in the hematopoietic organs: peripheral blood (PB), bone marrow (BM) and spleen (Spl). Genomic DNA was extracted from samples and used for PCR-amplification of the barcode-containing sequences. Sequencing was performed on a Miseq-Instrument and obtained sequences were analyzed by a customized R-script. Clone A is marked in green, clone B in blue, respectively

To investigate, whether these two clones were only successful by chance, we performed a second series of transplantations: 14 mice received 10,000 eGFP-positive cells from a second sort at day 49 of in vitro culture (compare Fig. 1). After 15 days, all mice showed signs of leukemia and were sacrificed. They all had enlarged spleens and eGFP-positive cells in all analyzed hematopoietic organs (PB, BM, spleen) (Additional file 1: Table S1 and Figure S4). Sequence analysis revealed that the majority of read counts in each mouse again represented the same two clones A and B, which were detected in the first cohort (Fig. 3). This time, however, both of the two clones were co-dominant in almost all animals simultaneously, even though with varying contributions. Analysis of the graft showed that both clones were already more abundant, as 20.4% of all reads represented barcode A, and 10.1% of all reads barcode B, indicating a certain growth advantage in vitro.

### Transcript level analysis

**Fig. 4:**
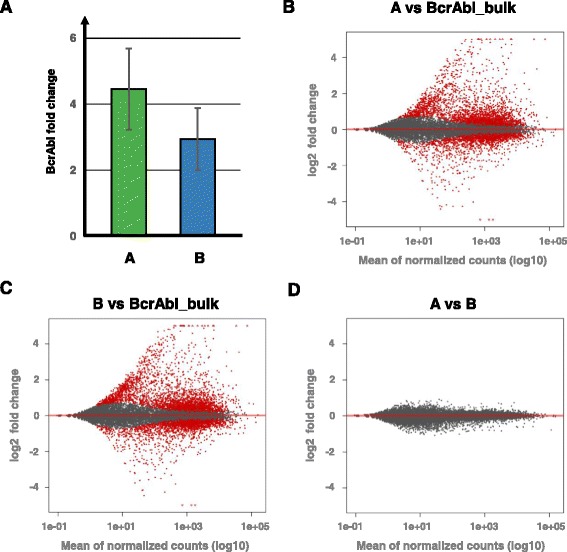
BcrAbl transcript levels and differential gene expression. **a** BcrAbl transcript levels were measured by quantitative real-time PCR (qPCR). BcrAbl transcript levels are increased in samples of clone A and B in comparison to samples of BcrAbl_bulk. **b** Differential expression among samples of clone A and BcrAbl_bulk; **c** among samples of clones B and BcrAbl_bulk and **d** among samples of clones A and B (**d**). DESeq2-normalized mean expression (x axis; log10 scale) and fold changes (y axis; log2 values) of differentially expressed (red) or unchanged (grey) genes (10% FDR) are indicated

Several studies indicate that the amount of BcrAbl transcript is a major determinant to define the transition from chronic phase CML to blast crisis (e.g. [40]). We established a qPCR method based on plasmid standards (used in clinical diagnostics) to assess the BcrAbl abundance in individual clones. In particular, we applied the ∆∆C_t_-method [41] to calculate the amount of BcrAbl transcripts in different BcrAbl clones in comparison to the sorted BcrAbl_bulk culture. We found that clone A contained 4.5× BcrAbl transcripts and clone B 2.9× more BcrAbl transcripts in comparison to the BcrAbl_bulk culture (Fig. 4a).

### Integration site analysis and transcriptomics

For further characterization of clones A and B, we performed ligation-mediated (LM-)PCR of DNA extracted from spleen of two diseased mice (clone A: mouse #11104 and clone B: mouse #11125). In accordance with the observed monoclonality we obtained one integration site (IS) for each sample. The IS of clone A was identified on chromosome 3 in the proximity of the tumor-suppressor gene *Sprouty1* (Gene ID: 24,063). Clone B harbored an IS on chromosome 13, localized downstream of the tumor suppressor *Klf-6* (Gene ID: 23,849). Both genes play a role in signaling pathways affected by BcrAbl. We therefore reasoned that a cooperative effect of the BcrAbl expression by dysregulation of genes close to the integration site might have contributed to the pronounced phenotype in vivo.

To further analyze the molecular basis of this clonal competition phenomena, we performed RNA-sequencing on 3 samples from both clones A and B and also 3 technical replicates of the controls. From the obtained reads (mean: 33 × 10^6^ reads per sample), we uniquely mapped on average 71.4% of all reads per sample (69.8–73.2%) to the mouse genome and analyzed the read counts for the tumor suppressor genes *Sprouty1* and *Klf-6*. Gene expression of *Sprouty1* (Spry1) was almost undetectable in all analyzed samples. On the contrary, *Klf-6* (Klf6) was reliably expressed in the non-transduced Ba/F3 cells with a 2- to 3-fold increase in clones A and B (Additional file 1: Figure S5). Together, this data indicates that vector insertions had no major impact on the expression of the two genes.

We analyzed the similarity in the gene expression profiles, and observed a distinct clustering of the replicates from clones A and B (based on Euclidian distance calculation) and a separation from the BcrAbl_bulk, which is depicted in the corresponding heatmap and further supported by principle component analysis (Additional file 1: Figure S6A and B). We identified 5755 genes that were differentially expressed in the clone A replicates (Fig. 4b), and 6556 differentially expressed genes in the clone B replicates (Fig. 4c) (FDR < 1). These genes were subjected to KEGG pathway analysis [42], to investigate possible mechanisms that might lead to the clonal success. We identified several pathways, some of which are affected in infection processes but also pathways relevant in signaling processes associated with cancer (Additional file 1: Figure S7). In contrast, we did not find any significant differences between clones A and B (Fig. 4d), thereby confirming the expressional similarity described above.

### Simulation studies

**Fig. 5:**
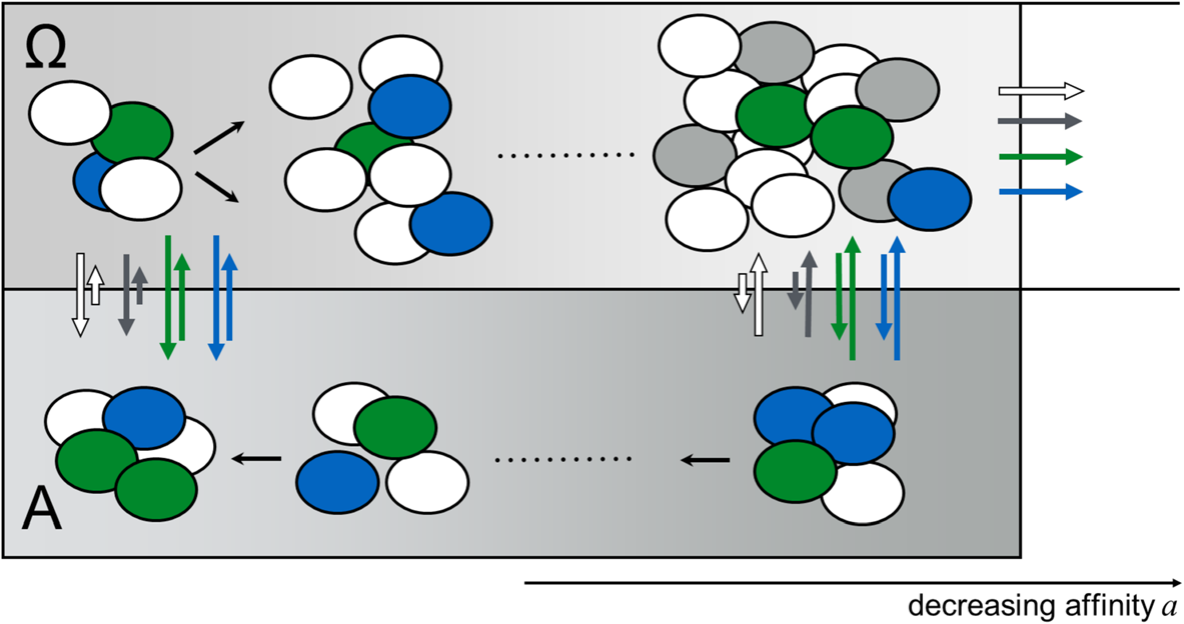
Model concept of clonal leukemia. Within the mathematical model normal (*white*) hematopoietic stem cells compete with leukemic (grey, blue, *green*) stem cells for niche space (lower, area A, no proliferation) in a stochastic activation/inactivation cycle (repeated changes to the proliferative stage (upper, area Ω). Among the leukemic cells, the green clone (“A”) and the blue clone (“B”) have a higher probability to occupy niche space (indicated by the size of the right vertical arrows) as compared to the other transformed Ba/F3 cells (BcrAbl_bulk indicated by the grey cells) or the host cells (white). The in silico cells are also able to differentiate indicated by the horizontal arrows (top right)

In order to investigate the mechanisms of clonal success, we performed simulation experiments with a single-cell based mathematical model of hematopoietic tissue maintenance [35, 36, 43] to test different assumptions about the intrinsic heterogeneity of the transplanted clones (Fig. 5).

**Fig. 6:**
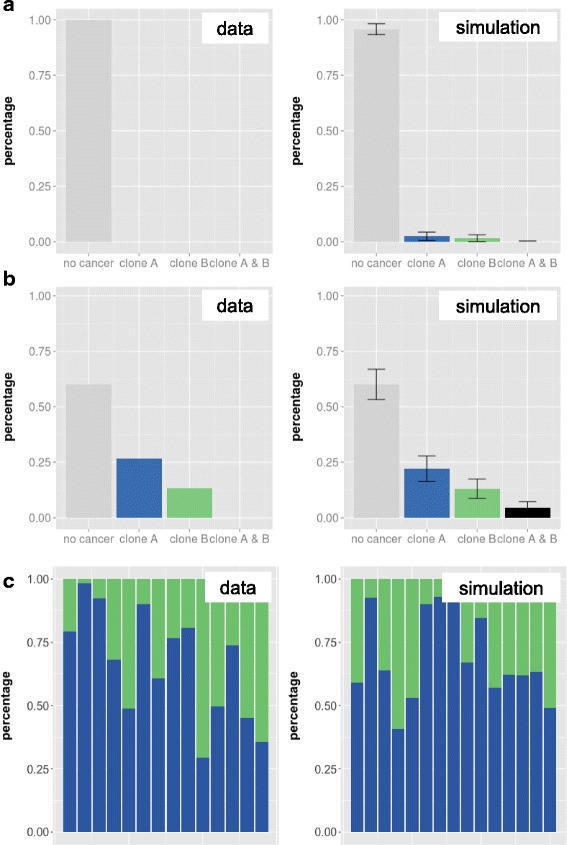
Comparison with simulation studies. Frequency of clones is compared between transplantation studies and the corresponding model simulations. **a**, **b** Percentages of recipients with no, monoclonal or biclonal leukemia for mouse transplantations with **a** 1000 and **b** 10,000 BcrAbl transduced cells at d29. Given the low initial frequency, no leukemic engraftment is shown for the cohort receiving 1000 cells while low numbers of monoclonally derived leukemias are observed in vivo and confirmed in silico for the cohort receiving 10,000 cells. **c** Composition of biclonal leukemias (consisting of contributions from clone A and B) for the second cohort of transplantation at d45. A corresponding composition is observed for the simulation studies

We assumed that cells show differences in their clonal proliferation ability or their competence to occupy hematopoietic niches and additionally, that the cells differ in their ability for engraftment. From our model we conclude, that a low overall engraftment is necessary to reflect the cell number dependence of leukemia formation. Under the proposition that clones A and B are structurally similar (as indicated by the parallel outgrowth in the second round of transplantations), the situation of either clone A *or* clone B dominating can only be reproduced if one but not the other clone engrafts. By the assumption that the engraftment efficacy is slightly better for clone A *and* clone B in comparison to all other transplanted cell clones, we were able to achieve a scenario that we observed empirically. Due to the sampling effect with the low numbers of transplanted cells, in most of the cases neither one of the two clones engrafted (Fig. 6a). If there was an engraftment, most often only one clone promotes the monoclonal leukemic outgrowth (Fig. 6b). Only an increase in absolute cell numbers of clones A and B within the prepared graft proved sufficient to explain not only the increase in leukemic mice but also to ensure a parallel engraftment of both leukemic clones (Fig. 6c).

Whether the dominance of clone A and B compared to the BcrAbl_bulk results solely from a superior engraftment or whether it is based on an additional proliferative advantage conferred by the in vivo situation, cannot entirely be disentangled based on the model structure.

## Discussion

In this work we investigated the clonal competition during leukemia outgrowth. To do so, we transduced murine Ba/F3 cells with barcoded γ- retroviral vectors, encoding the BcrAbl-oncogene. Thus we generated a BcrAbl_bulk population containing 158 different barcoded clones. Time course of in vitro cultures revealed fluctuations in clonal abundance, although the overall clonal composition only changed moderately over an observation period of 6 weeks. We observed a slow decline in clone numbers as described for regular cell culture [44]. However, we detected that the sorting of transduced cells had a distinct impact on clone numbers and clonal abundancies.

The clonal pattern changed once we transplanted eGFP-positive barcoded cells into non-conditioned mice. Six (of 15) animals transplanted with 10,000 transduced cells in the first cohort developed leukemia within 21–23 days. Barcode analysis revealed that the leukemias in those animals were monoclonal (Fig. 2), thereby indicating that a distinct clonal advantage promoted engraftment and outgrowth of defined cells only, whereas other clones were unable to contribute to leukemia development. Surprisingly, in the six leukemic mice only two different dominant clones were identified (clone A: in 4/6 animals, clone B: in 2/6 animals, Fig. 2). In a second experiment, all transplanted mice developed leukemia, which was not monoclonal as detected in the first cohort, but in most cases biclonal comprising both aforementioned clones A and B (Fig. 3). Together this data indicates that there was a threshold for the minimal numbers of cells from leukemia-inducing clones A and B that determined the experimental outcome. In fact, in the 1000 cells cohort of experiment 1, an estimated mean of only 5–6 cells from clones A and B were transplanted per animal, obviously insufficient to induce leukemia. In the 10,000 cells cohort of experiment 1, cell numbers were 10 times higher, and this number of approximately 100 (sum of A and B) cells was sufficient to cause leukemia in 6/15 (40%) animals. In the second experiment, the estimated number of cells representing clones A and B was again more than 10 times larger, which warranted leukemia outgrowth supported by both clones in all animals.

To further analyze the mechanisms leading to the clonal success in the two mouse experiments, we measured the BcrAbl transcript levels in spleen cells taken from leukemic mice and detected a higher abundance in both clones A and B (Fig. 4a). The elevated BcrAbl levels in clone A and B might have mediated their competitive advantage in the in vivo setting as suggested by earlier observations that BcrAbl expression increases with progression of CML from chronic phase to blast crisis [40, 45].

RNA sequencing revealed that the tumor suppressor genes in the genomic proximity of the proviral integrations (namely *Sprouty1* (clone A) and *Klf-6* (clone B)) did not show significantly differential expression compared to BcrAbl_bulk culture (Additional file 1: Figure S5). This led us to conclude that the integration itself did not substantially influence the functional behavior of both clones by activating cooperative oncogenes or downregulating tumor suppressors. However, looking at the overall gene expression profile, we revealed a large number of differentially deregulated genes (Fig. 4b-d) in clones A and B compared with BcrAbl_bulk culture, whereas we could not detect significant differences in gene expression profiles between clones A and B. Analyzing pathways that contained deregulated genes, we detected an abundance of pathways involved in disease responses, especially during the process of viral or bacterial infection. Typically, NFκB – a protein involved in multiple cellular responses – is present in all these pathways. NFκB is activated by the BcrAbl oncogene [46] and responsible in the BcrAbl-mediated transformation process [47, 48]. NFκB was also shown to be involved in the regulation of hematopoietic stem cell renewal and the interactions with the microenvironment of the hematopoietic niche [49, 50]. We reasoned that NFκB-driven responses might be a possible mechanism that translates the elevated BcrAbl levels found in clones A and B into a functional advantage of the corresponding cells. A correlation of BcrAbl expression with engraftment was also described by Krause et al. showing the dependence of BcrAbl-positive cells on CD44 or selectins for homing into the bone marrow [51, 52].

The higher BcrAbl expression in the two clones could possibly be explained by the location of the vector insertion in the genome, as there is a strong influence of the genomic surrounding on the expression strength of integrated retroviral vectors. Indeed, we have previously shown that expression strength may vary by a factor of four for single insertions [53], which might be explained by their position to *cis*- or *trans*-regulating elements in the nucleus [54].

Contrasting the moderate clonal conversion in the in vitro setting with the rapid monoclonal outgrowth of leukemia in vivo we investigated, which differentially regulated mechanisms are conceptually suited to recover the experimentally observed results. Using an established, single-cell based mathematical model [35, 36, 43] of hematopoietic self-maintenance we could only mimic the experimental observations, if we assumed a low overall engraftment probability for all transduced cells and an additional difference between clones A, B and the remaining clones with respect to either their proliferative capacity or their differential ability for initial engraftment. Addressing a potential proliferative difference, earlier results on the BcrAbl-mediated induction of autocrine IL-3 secretion showed no direct effect in BaF3 cells [27]. However, it was shown for other cells lines that such an effect can contribute to IL-3 independence [55, 56]. Although we do not observe an advantage for clones A and B under IL-3 deprivation in vitro conditions, we cannot rule out that this selective effect promotes a proliferative clonal advantage of these cells in recipient animals.

## Conclusions

Our findings represent a primary example to document that clonal competition is not an absolute process, but highly dependent on the environmental context. This notion is intuitively accepted and already established in the evolution of species. However, our exemplary study documents that similar mechanisms also act in cancer evolution, in which a broad spectrum of environmental cues can determine the clonal composition and the time scales of clonal dominance as well as the progression to leukemia. We demonstrate that the effect of a proliferative advantage might only lead to clonal conversion in the long run, whereas selective pressure (as exerted in a transplantation setting) can lead to rapid changes in the clonal repertoire. Similar processes are repeatedly discussed for the role of specific anti-cancer drugs promoting the outgrowth of resistant clones that were previously controlled in the polyclonal situation. As such, the acquisition of secondary mutations (e.g., driving increased BcrAbl transcription) or even changes in environmental factors (such as drug administration) might distinctly alter the competitive advantage of a cell clone, thereby accounting for accelerated leukemic expansion as observed during the transition from chronic phase to blast crisis. Our study thus represents an important step towards understanding the broadness of competition processes that lead to the manifestation of leukemia.

## Additional files

Additional file 1: Figure S1. eGFP expression and clonal kinetics in the BcrAbl_bulk culture. **Figure S2**: Overlap of barcodes from plasmid library and first sample. **Figure S3**: Clonal kinetics in the eGFP-positive cultures after sorting. **Figure S4**: Spleens from diseased (and control) animals. **Figure S5**: Read counts for Klf6 and Sprouty1. **Figure S6**: Comparison of samples. **Figure S7**: KEGG enrichment analysis. **Table S1**: Spleen weights and eGFP expression in the diseased animals. Supplementary Model Description. **Table S2**: Model Parameters. **Table S3**: Oligonucleotides. (PDF 7697 kb)

## Abbreviations

BC32: Barcode 32 construct
BM: Bone marrow
cDNA: Complementary desoxyribonucleic acid
CML: Chronic myeloid leukemia
DNA: Desoxyribonucleic acid
eGFP: Enhanced green fluorescent protein
FC: Flow cytometry
FDR: False discovery rate
IL-3: Interleukin 3
IS: Integration site
LM-PCR: Ligation-mediated polymerase chain reaction
PB: Peripheral blood
qPCR: Real-time quantitative polymerase chain reaction
RNA: Ribonucleic acid
Spl: Spleen
