# Supplementary material for: Clonal competition in BcrAbl-driven leukemia: how transplantations can accelerate clonal conversion
Source: Mol Cancer. 2017 Jul 14;16:120. doi: 10.1186/s12943-017-0668-x (PMC5512731; doi:10.1186/s12943-017-0668-x)
Supplement: Supplementary file 1 — eGFP expression and clonal kinetics in the BcrAbl_bulk culture. Figure S2: Overlap of barcodes from plasmid library and first sample. Figure S3: Clonal kinetics in the eGFP-positive cultures after sorting. Figure S4: Spleens from diseased (and control) animals. Figure S5: Read counts for Klf6 and Sprouty1. Figure S6: Comparison of samples. Figure S7: KEGG enrichment analysis. Table S1: Spleen weights and eGFP expression in the diseased animals. Supplementary Model Description. Table S2: Model Parameters. Table S3: Oligonucleotides. (PDF 7697 kb) [file 12943_2017_668_MOESM1_ESM.pdf]

Additional file

Clonal competition in BcrAbl-driven leukemia: a mathematical model reveals mechanisms of clonal conversion

Kerstin Cornils, Lars Thielecke, Doreen Winkelmann, Tim Aranyossy, Mathias Lesche, Andreas Dahl, Ingo Roeder, Boris Fehse, Ingmar Glauche

Figure S1

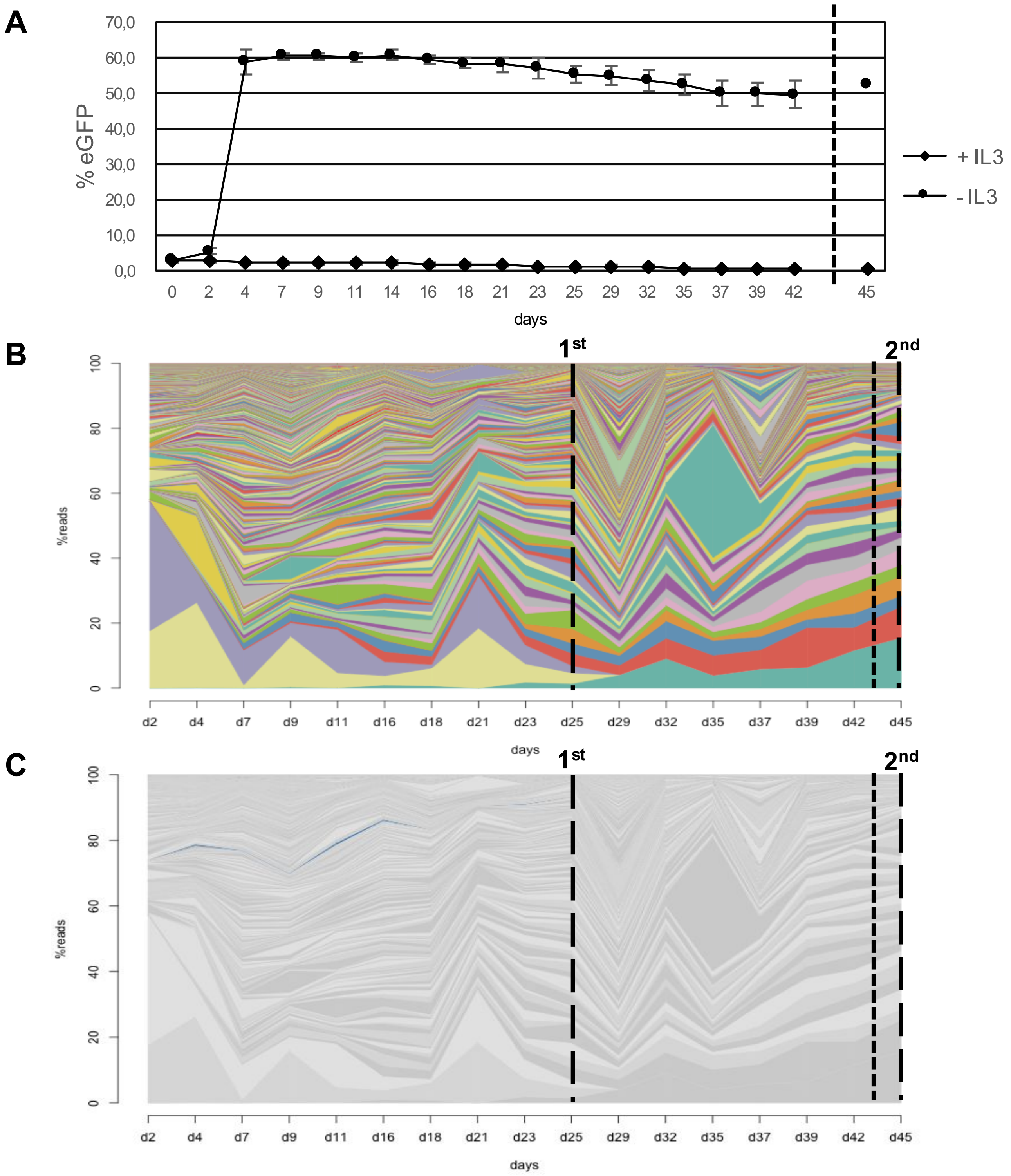

Figure S1 : eGFP expression and clonal kinetics in the BcrAbl-bulk culture

(**A**) After IL-3 deprivation, we followed the eGFP expression of the BcrAbl culture over 42 days (dots) in comparison to cells kept in IL-3 containing medium (rectangles). The expression remained almost stable over time and did not change after a freeze-thawing process (dotted line). (**B**) The clonal kinetics of the IL-3-deprived bulk culture showed a loss of clones over time (starting with 158 clones, declining to 114 clones). The freeze-thawing process after day 42 did not lead to a clonal conversion (dotted line). On day 25 and day 45, eGFP-positive cells were sorted from the bulk culture (dashed lines). (**C**) The two clones, dominating the leukemias in the transplanted mice are marked in blue and green. They were present at less than 0.5% during culturing.

Figure S2

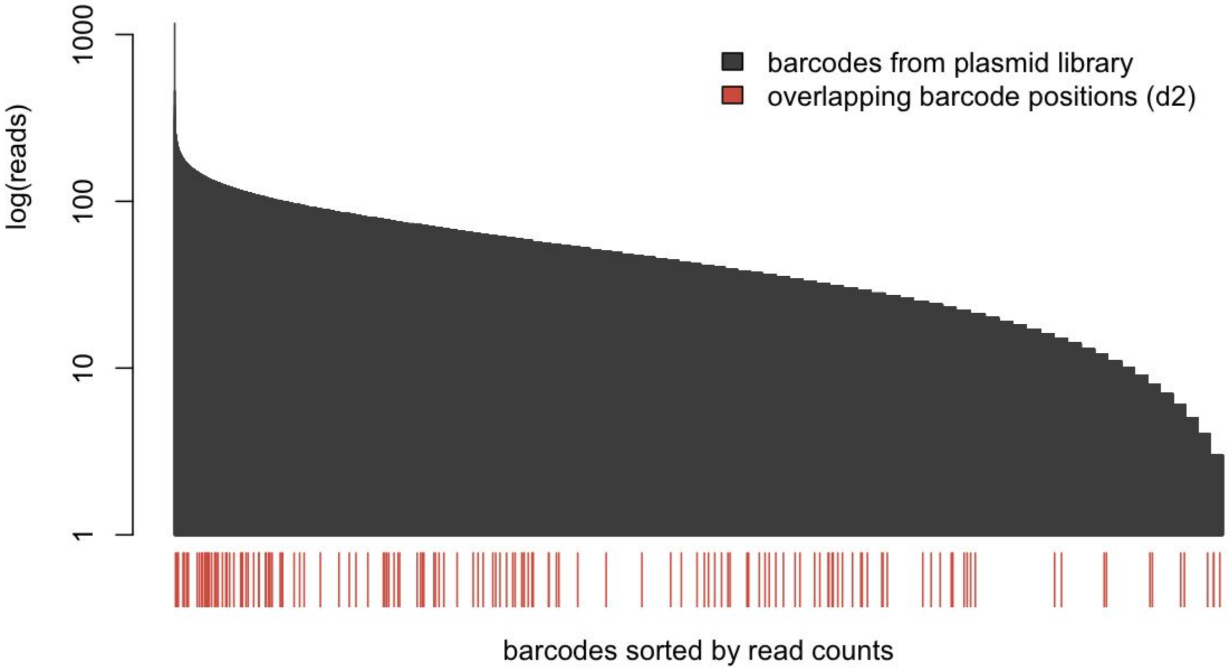

Figure S2: Overlap of barcode from plasmid library and first sample

Distribution of log-transformed read counts of all barcodes within the sequenced plasmid library. Overlapping barcodes from plasmid library and first sample (d2) are depicted in red / mirrored bars, illustrating the almost equal distribution over all plasmid barcodes in terms of initial read counts.

Figure S3

A

1st Sort:

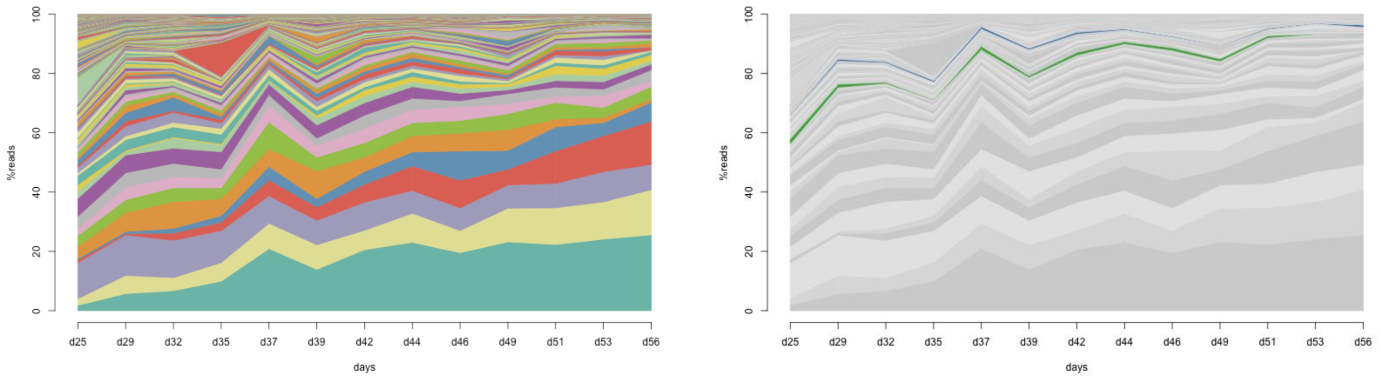

B

2nd Sort:

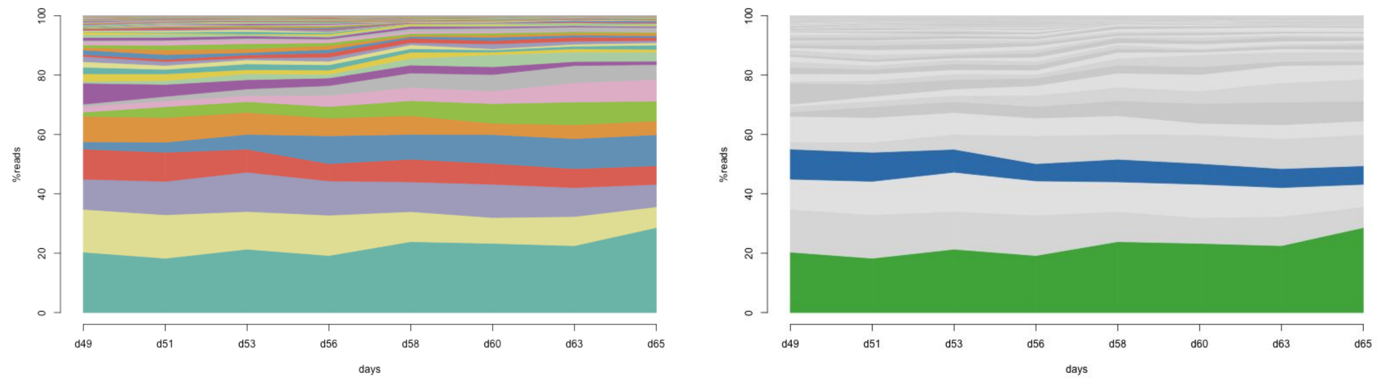

Figure S3: Clonal kinetics in the eGFP-positive cultures after sorting

On d25 and on d45, we sorted the BcrAbl bulk into eGFP-positive and eGFP-negative cells. We followed the clonal dynamics in the eGFP-positive cultures over time. (A) eGFP-positive cells after the first sort and (B) after the second sort. The right panels highlight the two clones being dominant in the mouse transplantation experiments.

Figure S4

First Transplantation d29:

A

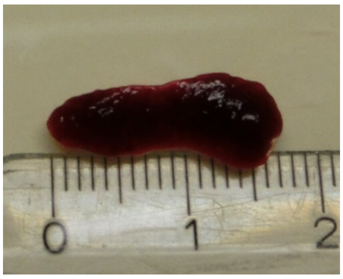

Control Animal
(133 mg)

B

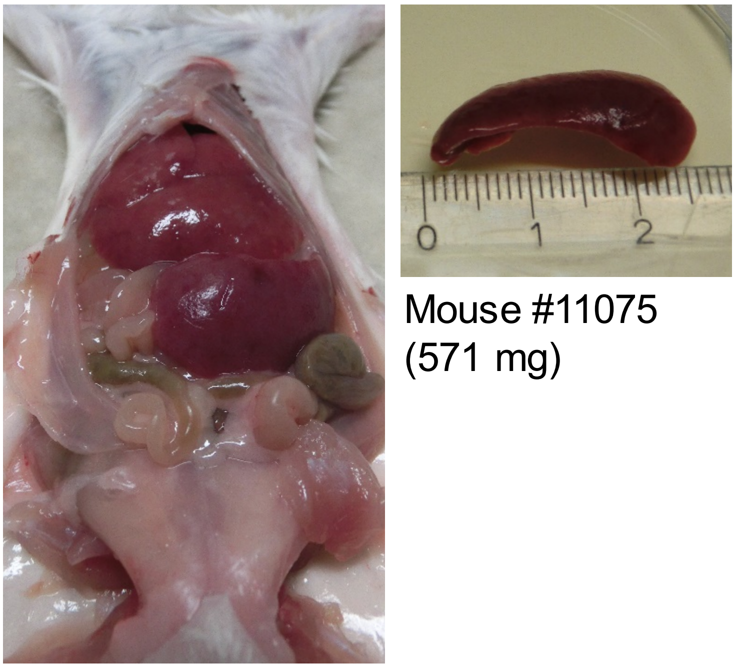

Mouse #11075
(571 mg)

Second Transplantation d49:

C

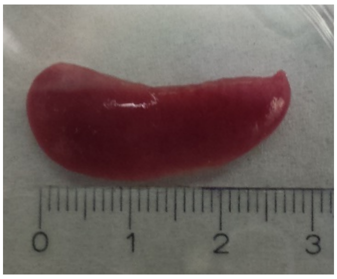

Mouse #1756
(801mg)

D

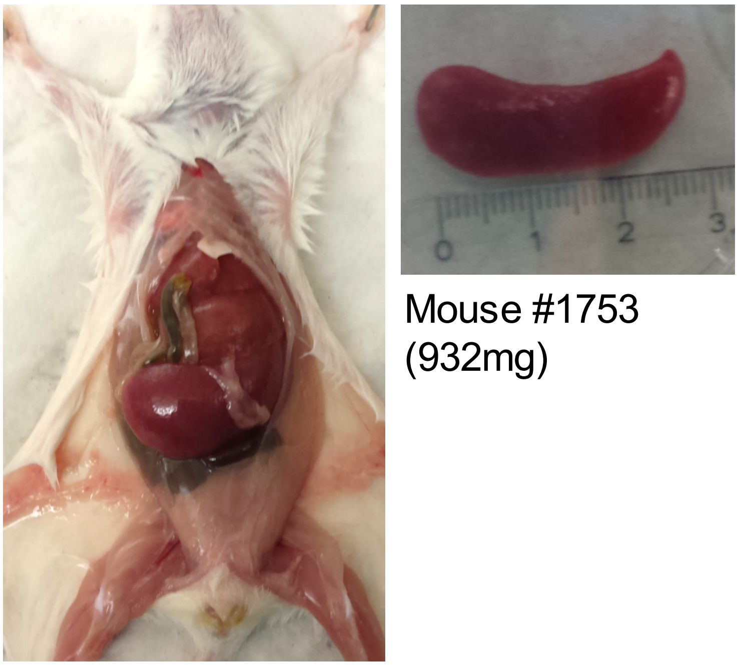

Mouse #1753
(932mg)

Figure S4: Spleens from diseased (and control) animals
(**A**) Spleen taken from a control animal. (**B**) Autopsy of animal #11075 from the first transplantation experiment from d29 showed an enlarged spleen. (**C**) Spleen taken from animal #1756. (**D**) Autopsy and spleen of taken from animal #1753 of the second transplantation. Spleens taken from animals of the second transplantation experiment were also enlarged.

Figure S5

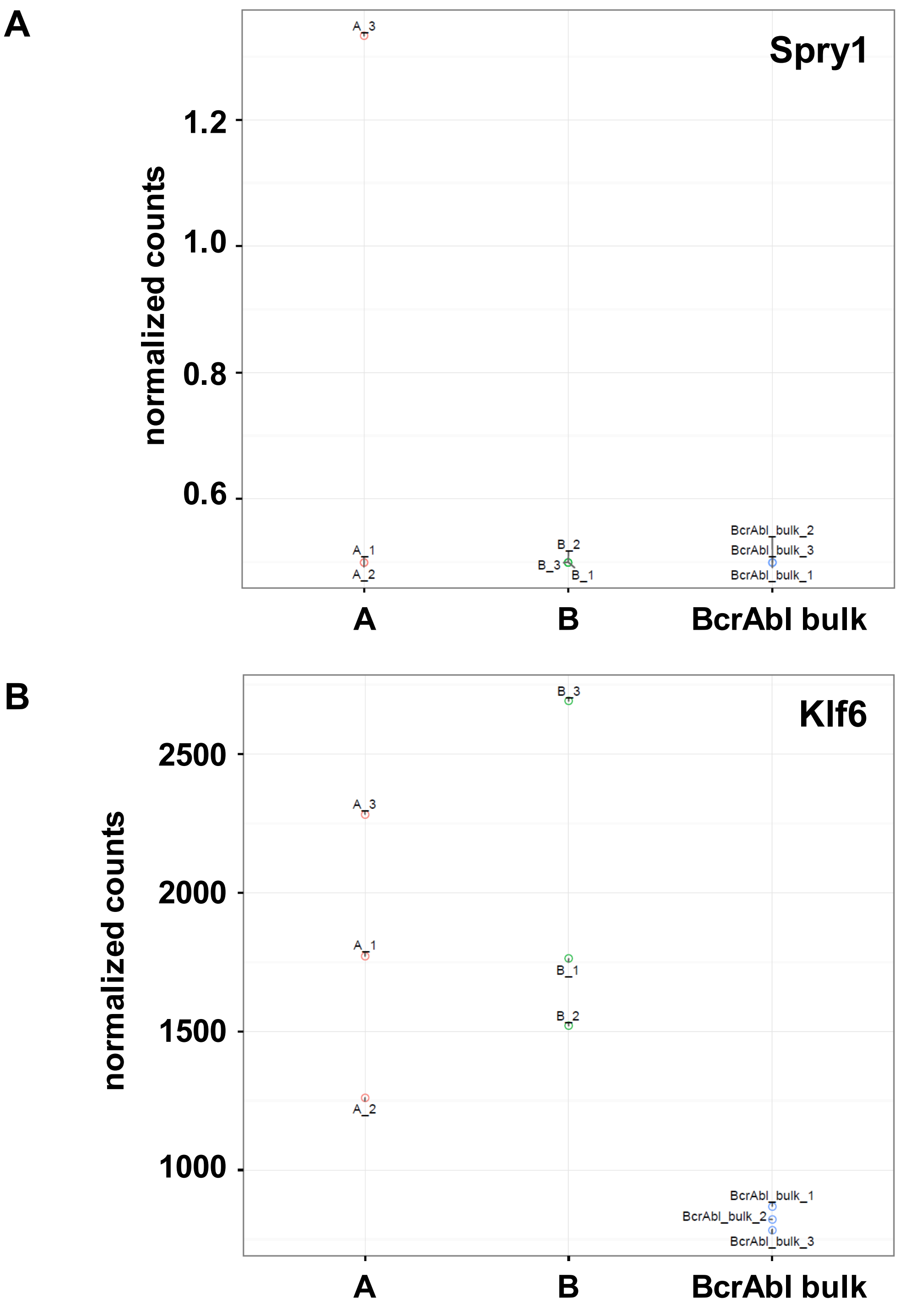

Figure S5
(**A**) Normalized read counts for the gene *Sprouty1 (Spry1)*, located in the proximity to the viral integration site of clone A. There are only a few reads present from the gene in the sample. (**B**) Normalized read counts for the genes Klf-6, located in the proximity to the viral integration site of clone A. In comparison to the BcrAbl bulk we detected increased numbers of reads in clone B, but also in clone A.

Figure S6

A

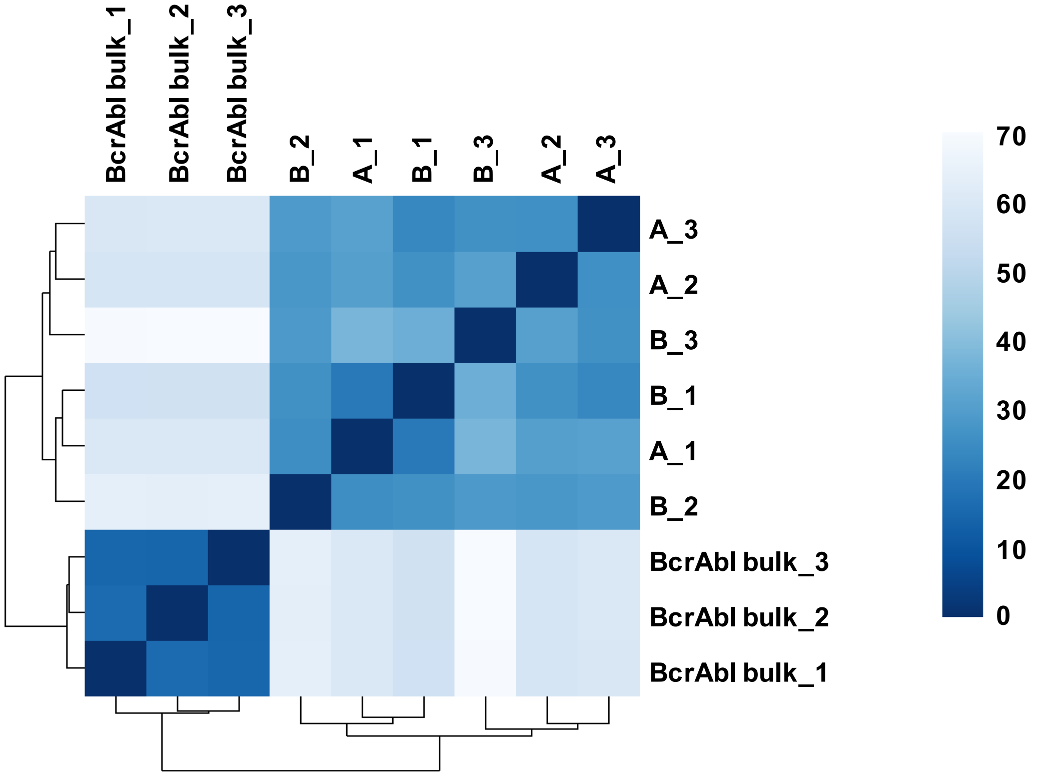

B

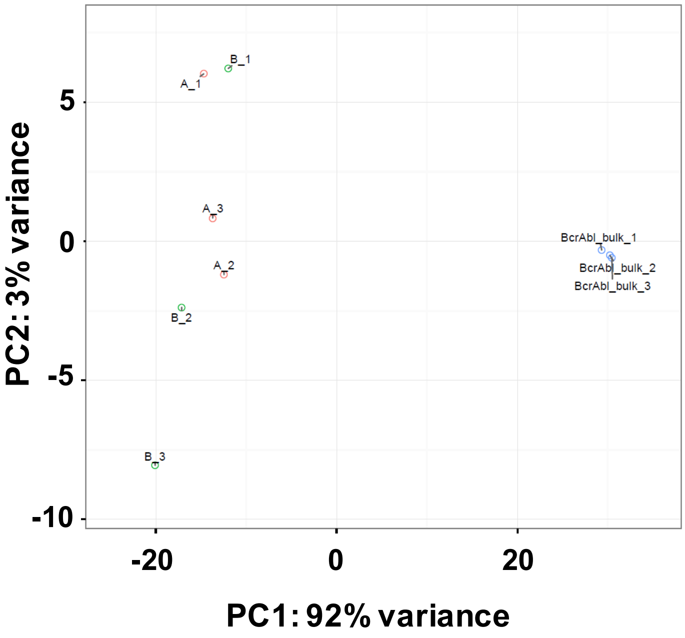

Figure S6: Comparison of samples

(**A**) Sample to sample correlation based on calculation of the Euclidian distance of DESeq2-normalized gene expression between three replicates of clone A, B and the BcrAbl bulk. (**B**) Principle component analysis of the TOP 500 most diverse genes.

Figure S7

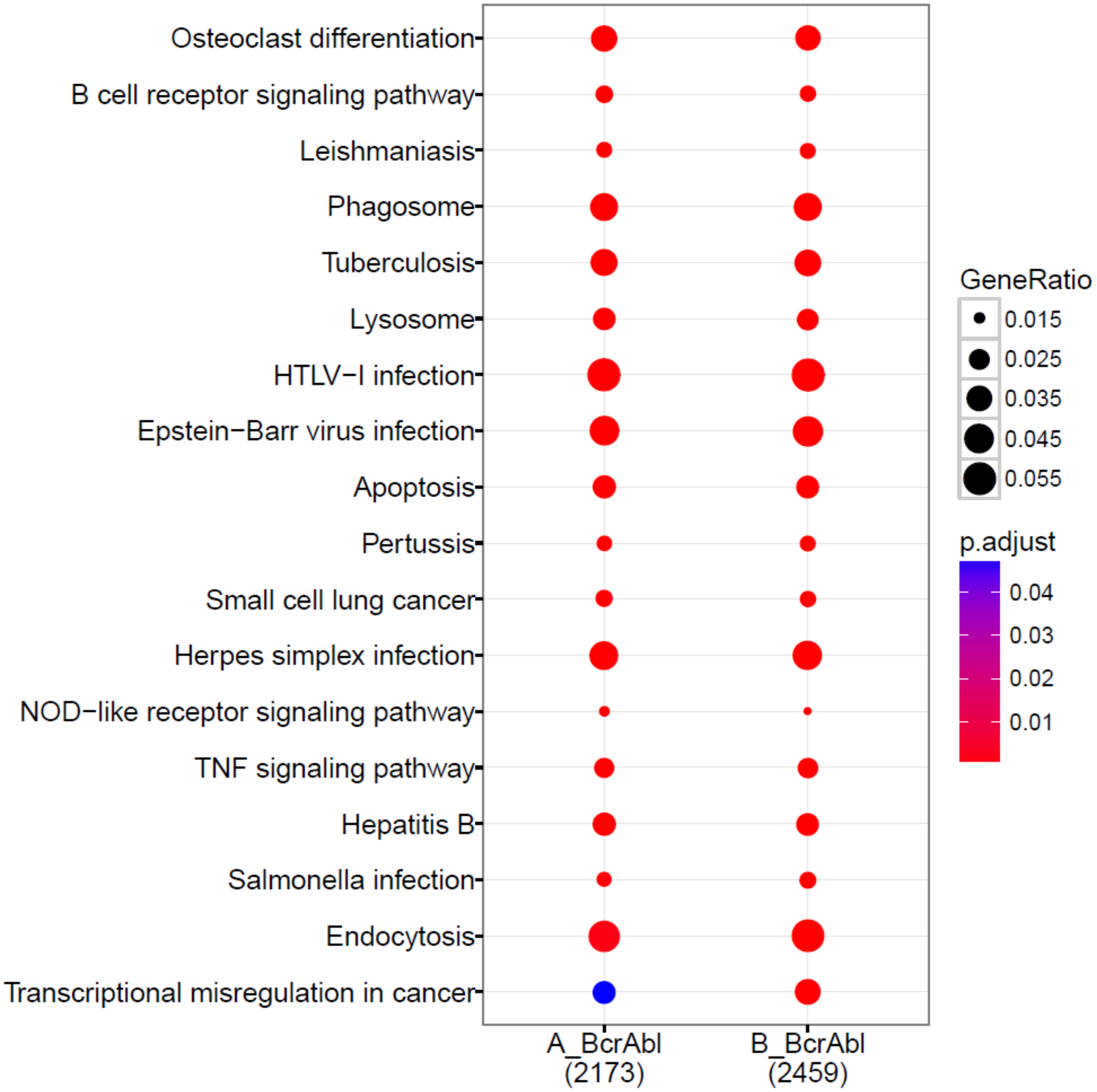

Figure S7: KEGG enrichment analysis

KEGG gene set enrichment analysis was performed with the clusterProfiler R package (Yu et al. 2012). On the X-axis, the two comparisons and their significantly differentially expressed genes (in brackets) are shown. On the y-axis the differentially regulated pathways are shown. The size of the circles indicates how many genes of the significant genes are involved the corresponding pathway. p-adj value shows the significance of a particular pathway.

Table S1: Spleen weights and eGFP expression in the diseased animals

6 mice from the first transplantation experiment (I.; day 29) of the 15 animals which received 10,000 cells showed signs of disease on day 21 or 23 after transplantation and were sacrificed. From the second transplantation experiment (II.; day 49), all mice were sacrificed on day 15 after transplantation, when the animals showed signs of disease. Spleens were mostly enlarged and eGFP-expressing BaF/3 cells were detected by flow cytometry in the peripheral blood, bone marrow and in the spleen.

Experiment	mouse #	spleen weight (mg)	% GFP in		
			periph. blood	bone marrow	spleen
	**11104**	1026	31.5	20.1	51.2
	**11076**	1094	30.1	11.6	51.9
**I.**	**11073**	800	12.8	1.2	26.7
**(d29)**	**11075**	571	16.6	5.0	24.8
	**11077**	952	11.6	0.7	25.0
	**11125**	311	4.9	15.5	11.3
	**1754**	911	13.9	19.7	36.6
	**1756**	801	2.2	15.1	40.5
	**1757**	572	14.0	7.4	23.1
	**1748**	180	1.4	9.7	0.6
	**1744**	685	11.9	7.8	21.6
	**1749**	464	5.3	5.9	23.6
**II.**	**1751**	811	17.7	9.2	36.1
**(d49)**	**1752**	713	16.8	10.1	26.8
	**1753**	932	23.8	21.1	40.3
	**1755**	645	17.6	8.3	26.4
	**1745**	733	13.9	15.3	16.3
	**1746**	590	22.4	11.0	23.2
	**1747**	350	0.2	5.1	7.8
	**1750**	205	0.5	4.3	0.3

Supplementary Model Description

The mathematical model (Roeder and Loeffler, 2002; Roeder et al, 2005) explains leukemogenesis as a competition process between normal and leukemic cells, in which a proliferative advantage of the leukemic cells leads to a sustained outcompetition of normal hematopoiesis. In particular, the model assumes that both normal stem cells and transduced leukemic clones reside in either of the two signaling contexts, named A and Ω, and that they can reversibly change between them (Figure S9). Importantly, the signaling contexts impose different effects on the cellular development: whereas context A is inspired by the concept of a supporting niche which promotes cellular quiescence and regeneration, context Ω represents an escape from the niche signals and promotes proliferation and differentiation. The tendency of a cell to switch from one context to the other is determined by the cell number within the target compartment, implemented as a compartment-specific sigmoid activation/deactivation function fα/ω and by a cell-specific affinity a to reside in context A. The affinity a is gradually lost in context Ω, but regained in A up to the maximum value a_{max}. Therefore, the system is able to dynamically establish and retain an equilibrium, balancing quiescent cells in A and proliferating cells in Ω.

For the simulation of the transplantation setting we start with an undisturbed system of normal cells mimicking the host individual under homeostatic conditions. Depending on the clonal origin of the leukemic clone (A or B, shown in green and blue), single cells are sampled from a distribution of affinity values (Supplementary Table 2), for which a small fraction of cells has values above the threshold and can potentially engraft. Clonal expansion results from the proliferative advantage once the leukemic clone has engrafted. We score a simulation as “leukemic” if any or several of the transplanted clones are retained and outcompete the normal cells.

Table S2: Model Parameters refer to the sigmoid transition functions between the compartments A and Ω, and which differ between normal (A) and transformed (B) cells. Further details of the model implementation can be found in Roeder et al. 2006, Horn et al. 2013, or Glauche et al. 2012.

		f(0)	f(N/2)	f(N)	f(∞)	Nnorm
A	**fα**	0.5	0.3	0.01	0	1300
	**fω**	0.5	0.0075	0.0002	0	280
B	**fα**	0.8	0.72	0.062	0	1300
	**fω**	0.8	0.0625	0.00625	0	280

Table S3: Oligonucleotides

Function	Name	Sequence (5'->3')
**Generation of vector backbone**		
BC-MCS	32BCgMCSfw	AGGTTAATGATACGGCGACCACCGCAATTGTATAGCGCTATAGACGCGTAGATCGGAAGAGCACAAGTCTGAACTCCAGTCACA
	32BCgMCSrv	AGCTTGTGACTGGAGTTCAGACTTGTGCTCTTCCGATCTACGCGTCTATAGCGCTATACAATTGCGGTGGTCGCCGTATCATTA
**Barcode generation**		
Generation of BC32	32BCgOligoMfe	GGGCAATTGACACTCTTTCCCTACACGACGCTCTTCCGATCTNNNACTNNCGANNCTTNNCGANNCTTNNGGANNCTANNACTNNCGANNCTTNNCGANNCTTNNGGANNCTANNACTNNCGANNACGCGT
	32BCg-Poly_rv	CATAGTGCACCTCGAG
**Barcode amplification**		
BC32 barcode retrieval	MPLX-primer	CAAGCAGAAGACGGCATACGAGATxxxxxxGTGACTGGAGTTC
	p43	GTGACTGGAGTTCAGACGTGTGCTCTTCCGATCT
	DUAL-Index-primer	AATGATACGGCGACCACCGAGATCTACACxxxxxxxxACACTCTTTCCCTACACGACGCTCTTCCGATCxT
**quantitative Real-time PCR**		
house keeper	mGAPDH-(PS)-FW	TGTCAAGCTCATTTCCTGGTATGA
	mGAPDH-(PS)-pro	HEX-CCCAACTCGGCCCCCAACACTG-TAMRA
	mGAPDH-(PS)-RV	CTGTTATTATGGGGGTCTGGGT
BcrAbl	ENF402*	CTGGCCCAACGATGGCGA
	ENP541*	FAM-CCCTTCAGCGGCCAGTAGCATCTGA-TAMRA
	ENR561*	CACTCAGACCCTGAGGCTCAA

* Published by Gabert et al., 2003, Leukemia; 17:2318-57
